# Supplementary material for: Initial phospholipid-dependent Irgb6 targeting to Toxoplasma gondii vacuoles mediates host defense
Source: Life Sci Alliance. 2019 Dec 18;3(1):e201900549. doi: 10.26508/lsa.201900549 (PMC6925386; doi:10.26508/lsa.201900549)
Supplement: Supplementary file 1 [file LSA-2019-00549_TableS1.doc]

Table S1. Primer list used in this study (next page)

| Primer name | Enzyme | Sequence | Resulting plasmids and descriptions |
| --- | --- | --- | --- |
| WT_Irgb6_full_F | EcoRI | 5’-gaattcaccATGGCTTGGGCCTCCAGCTTTGA-3’ | pMRX vector |
| WT_Irgb6_full_R | XhoI-Stop-NotI | 5’-gcggccgctactcgagAGCTTCCCAGTACTCGGGGGGCTCA-3’ | pMRX vector |
| K69A_F | ー | 5’-GAAACAGGCGCAGGGgcGTCCACTTTCATCAATACC-3 | pMRX vector |
| K69A_R | ー | 5’-GGTATTGATGAAAGTGGACgcCCCTGCGCCTGTTTC-3’ | pMRX vector |
| S70N_F | ー | 5’-ACAGGCGCAGGGAAGaaCACTTTCATCAATACCCTG-3’ | pMRX vector |
| S70N_R | ー | 5’-CAGGGTATTGATGAAAGTGttCTTCCCTGCGCCTGT-3’ | pMRX vector |
| K233A_F | ー | 5’-CTTCCCAAAGCTGGAAACTgcACTCCTACAGGATCT-3’ | pMRX vector |
| K233A_R | ー | 5’-AGATCCTGTAGGAGTgcAGTTTCCAGCTTTGGGAAG-3’ | pMRX vector |
| K266A_F | ー | 5’- AAGAGAGATTCCCTGgcGCAAAAAGTCTTCCTA-3’ | pMRX vector |
| K266A_R | ー | 5’-TAGGAAGACTTTTTGCgcCAGGGAATCTCTCTT-3’ | pMRX vector |
| K268A_F | ー | 5’-GAGAGATTCCCTGAAGCAAgcAGTCTTCCTAGAAGCC-3’ | pMRX vector |
| K268A_R | ー | 5’-GGCTTCTAGGAAGACTgcTTGCTTCAGGGAATCTCTC-3’ | pMRX vector |
| K275A_F | ー | 5’- AGTCTTCCTAGAAGCCATGgcGGCTGGAGCATTAGCC-3’ | pMRX vector |
| K275A_R | ー | 5’- GGCTAATGCTCCAGCCgcCATGGCTTCTAGGAAGACT-3’ | pMRX vector |
| R371A_F | ー | 5’-GCTGCAGTCACTTACTATgcCATGGCTTATTATTTGCAG-3’ | pMRX vector |
| R371A_R | ー | 5’-CTGCAAATAATAAGCCATGgcATAGTAAGTGACTGCAGC-3’ | pMRX vector |
| K395A_F | ー | 5’-CATAGCTCTTCTGAATAGTgcAGCACTTTTTGAGAAG-3’ | pMRX vector |
| K395A_R | ー | 5’-CTTCTCAAAAAGTGCTgcACTATTCAGAAGAGCTATG-3’ | pMRX vector |
| Delta340-415_R | XhoI-Stop-NotI | 5’-gcggccgctactcgagTTCTGCAAACAAATGGGGAAATCGAAG-3’ | pMRX vector |
| Delta250-415_R | XhoI-Stop-NotI | 5’-gcggccgctactcgagAGACAGTGAGAATACGTGACGCTTGTG-3’ | pMRX vector |
| mIrgb6_gRNA1_F |  | 5’- TTA ATA CGA CTC ACT ATA GGt gtg ggc tgg gag atc ctg tGT TTT AGA GCT AGA AAT AGC AAG TTA AAA T-3’ | Generation of Irgb6 KO mice |
| mIrgb6_gRNA2_F |  | 5’- TTA ATA CGA CTC ACT ATA GGt ctg cac ata gct gtg aca gGT TTT AGA GCT AGA AAT AGC AAG TTA AAA T-3’ | Generation of Irgb6 KO mice |
| mIrgb6*_gDNA_F |  | 5’-CTCTAAGCCAAAGGAAACCAGAAGT-3’ | Genomic DNA amplification  and sequencing |
| mIrgb6*_gDNA_R |  | 5’-GAGGAGAAAAATGCAAACCAAATCCA-3’ | Genomic DNA amplification  and sequencing |
| mIrgb6_gDNA_F |  | 5’-GTAAGACAGTCTAATAAGGCTAATAAGGTCG-3’ | Genomic DNA amplification  and sequencing |
| mIrgb6_gDNA_R |  | 5’-GAGGAGAAAAATGCAAACCAAATCCG-3’ | Genomic DNA amplification  and sequencing |
| mIrgb5-b4_cDNA_F |  | 5’-ATGGGTCAGACTTCCTCTTCTACAC-3’ | cDNA amplification and sequencing |
| mIrgb5-b4_cDNA_R |  | 5’-TCACAGCTCCATTCCTGTTTCCCAGTATTC-3’ | cDNA amplification and sequencing |
| mIrgb4-3' UTR-F |  | 5’-GTTGCTGATGCTCGAATCTATGGTTCCTGA-3’ | Genomic DNA amplification |
| mIrgb4-3' UTR-R |  | 5’-CAACACATCTTCTGGGACAGGCCCTGCTTTGG-3’ | Genomic DNA amplification |
